# *Idiomarina solivarensis* sp. nov., halophilic bacterium isolated from brine of the former subsurface salt mine in Solivar (Slovakia) showing a unique fatty acids profile

**DOI:** 10.1007/s00203-026-05004-3

**Published:** 2026-06-16

**Authors:** Soňa Brestovičová, Lea Nosáľová, Jana Kisková, Lukáš Malčický, Peter Pristaš

**Affiliations:** 1 Department of Microbiology, Institute of Biology and Ecology, Faculty of Science, Pavol Jozef Šafárik University in Košice, Šrobárova 2, 041 54 Kosice, Slovakia; 2 Present Address: Sorbonne Université, Université Paris Cité, Univ Paris Est Créteil, CNRS, IRD, INRAE, Institut d’écologie et des sciences de l’environnement de Paris, IEES Paris, 75005 Paris, France; 3 Department of Cell Biology, Institute of Biology and Ecology, Faculty of Science, Pavol Jozef Šafárik University in Košice, Šrobárova 2, 041 54 Kosice, Slovakia; 4 Institute of Animal Physiology, Centre of Biosciences, Slovak Academy of Sciences, Šoltésovej 4-6, 040 01 Kosice, Slovakia

**Keywords:** Halophilic bacteria, Brine, Salt mine, *Idiomarinaceae*, *Idiomarina*

## Abstract

Strain HP20-50^T^ was isolated from brine of a former salt mine in Solivar (Prešov, Slovakia). The cells are catalase- and oxidase-positive, aerobic Gram-negative short rods. Optimal growth occurred at temperature of 25 °C, pH of 7.0, and a salinity range of 3.0% to 6.0% NaCl (*w/v*). Phylogenetic analysis based on the 16 S rRNA gene sequences affiliated the strain with the genus *Idiomarina*, with the highest similarity to *Idiomarina ramblicola* R22^T^ (99.6%), followed by *Idiomarina loihiensis* L2TR^T^ (99.5%). Comparative whole-genome analyses of strain HP20-50^T^ with its closest related type strains of the genus *Idiomarina* showed average nucleotide identity (ANI) values ranging from 65.0% to 82.8%, average amino acid identity (AAI) values from 67.1% to 91.2%, and digital DNA–DNA hybridization (dDDH) values from 18.2% to 23.2%. The major fatty acids were C_11:0_ iso 3-OH (20.1%), C_13:0_ iso 3-OH (15.5%), C_11:0_ iso (10.0%), and C_12:0_ 3-OH (9.0%), with 3-OH fatty acids predominance, a pattern never observed in *Idiomarina* species neither other halophilic bacteria. Strain HP20-50^T^ has a genome DNA G + C content of 46.8% and a genome size of 2.8 Mb. Based on the genomic analyses and the unique phenotypic properties of strain HP20-50ᵀ, we propose that the strain represents a novel species of the genus *Idiomarina*, for which the name *Idiomarina solivarensis* sp. nov. is proposed. The type strain is HP20-50^T^ (= DSM 120110^T^ = CCM 9475^T^).

**Supplementary Information:**

The online version contains supplementary material available at https://doi.org/10.1007/s00203-026-05004-3.

## Introduction

Representatives of the genus *Idiomarina* are halophilic bacteria belonging to class *Gammaproteobacteria*. They are mostly found in marine habitats, such as seawater, seabed sediments and coastal wetlands (Albuquerque and da Costa 2014). *Idiomarina*-affiliated strains are classified as euryhaline, showing the ability to grow at a broad salinity range (0.5–20.0% *w/v*) (Taborda et al. 2009). For example, *Idiomarina seosinensis* thrives in the hypersaline environment of solar saltern (29.0–30.0% *w/v*) in South Korea (Choi and Cho 2005), whereas *Idiomarina baltica* showed growth at salinity levels as low as 0.8% (*w/v*) (Brettar et al. 2003).

Distinctions from other genera of the family *Idiomarinaceae* arise from the genetic and genomic differences, making the comparative genomics one of the main steps in the characterization of *Idiomarina*-affiliated strains (Liu et al. 2018). A dominant feature of the genus *Idiomarina* is its strongly reduced genome (Taborda et al. 2009; Brestovičová et al. 2024), especially with regard to genes involved in carbohydrate transport and metabolism. Conversely, from the genome analyses is apparent that they evolved the ability to use exogenous proteinaceous substrates as carbon and energy resources (Qin et al. 2019). Therefore, *Idiomarina* spp. primarily rely on amino acid catabolism in lieu of sugars fermentation, as a result of their adaptation to mostly oligotrophic conditions. Their DNA G + C content ranges from 45% to 54%, and their cell membranes typically contain iso-branched fatty acids with 15 and 17 carbons as the predominant components (Hintersatz et al. 2025).

Despite the growing number of described species within the genus *Idiomarina*, the adaptive mechanisms enabling survival in extremely hypersaline conditions remain poorly understood, particularly with regard to membrane adaptation and chemotaxonomic diversity. Previous studies have focused primarily on phylogenetic classification, genome reduction, and the enzymatic potential of marine representatives (Zhou et al. 2018; Qin et al. 2019; Kaur and Kaur 2024), while relatively little attention has been paid to strains originating from terrestrial hypersaline environments. Despite that the remodelling of membrane fatty acids may be driven by stress (García-Descalzo et al. 2023; Maiti et al. 2024), the unusual fatty acid composition of the membranes probably associated with adaptation to high salinity has not yet been studied within the genus *Idiomarina*. The characterization of new isolates of the genus *Idiomarina* from extremely saline habitats may therefore provide further insights into the ecological and physiological adaptation strategies of halophilic bacteria.

The interest in the genus *Idiomarina* research has expanded in recent years, particularly with regard to its biotechnological potential. They are well-known for productions of enzymes with interesting activities, such as proteases and/or lipases, exhibiting stability and activity under high salinity and over a wide range of pH and temperatures (Zhou et al. 2018; Kaur and Kaur 2024).

In this study, we present the detailed characterisation of the novel strain HP20-50^T^. This strain was discovered during the diversity analysis of the highly adapted microbiota of brine (Brestovičová et al. 2024) emerging from former subsurface salt mine in Slovakia (48.983529 N, 21.283090 E). Based on the 16 S rRNA gene sequences and genome analyses strain HP20-50^T^ was selected for novel species description, showing the clear relatedness to genus *Idiomarina*, but with unique fatty acids profile.

## Materials and methods

### Isolation and cultivation analyses

The strain HP20-50^T^ was isolated from brine of a former salt mine in Solivar (Prešov, Slovakia). The site is characterized by high salinity (~ 311 g/L TDS) and represents a terrestrial hypersaline environment. Detailed physicochemical characterization of the site and a map of the sampling location were previously published by Brestovičová et al. (2024). Briefly, the sample was collected at the water emergence from borehole into sterile 500 mL bottles, transported to the laboratory, and processed immediately. Then 100 µL of the water sample was used for the primary isolation, and inoculated onto R2A medium supplemented with 5.0% of NaCl (*w/v*) (Merck KGaA, Germany), cultivated at 25 °C under aerobic conditions until well-separated colonies were observed (24–48 h). Colonies were picked, and the bacterial strains were sub-cultured several times using the streak-plate technique, and subjected to further analyses. Stock cultures were stored in 50.0% of glycerol (*v/v*) (CentralChem, Slovakia) at -70 °C. The strain was deposited at the Czech Collection of Microorganisms (CCM) and at the Leibniz-Institut DSMZ - German Collection of Microorganisms and Cell Cultures GmbH in Germany under the accession numbers: CCM 9475^T^ and DSM 120,110^T^, respectively.

### Morphological analyses

Colony morphology of strain HP20-50^T^ was observed on R2A medium supplemented with 5.0% of NaCl (*w/v*) after two days of cultivation at 25 °C under aerobic conditions, using a Leica EZ4D stereo microscope (Leica Microsystems, Singapore). Gram staining was performed using a Gram Stain Kit TM (BD Biosciences, USA). Sporulation activity was determined using Schaeffer–Fulton staining after 48 h of cultivation on R2A medium (Sigma-Aldrich, USA) supplemented with 5.0% of NaCl (*w/v*), using 5% malachite green (Merck KGaA, Germany) and carbol fuchsin (Difco, USA). Cell morphology was examined using transmission electron microscopy, briefly an overnight bacterial culture (OD_600_ 0.6–0.8) was inoculated onto R2A medium supplemented with 5.0% of NaCl (*w/v*). After 12 h of cultivation, cells were gently washed with physiological saline, collected, and subsequently applied onto formvar-coated slot copper grids and negatively stained using 2% uranyl acetate (*w/v*) and inspected using a JEM 1230 transmission electron microscope (JEOL, Japan) at an accelerating voltage of 80 kV.

### Phenotypic characterization

Cultivation analyses were performed in TSB medium (Merck KGaA, Germany) supplemented with 5.0% of NaCl (*w/v*), unless otherwise stated. The temperature range for growth was determined at 4, 10, 15, 20, 25, 30, 37 and 40 °C. The optimum salinity was tested at NaCl concentrations ranging from 1.0 to 20.0% (*w/v*) with 1% increments, and growth at different pH values was tested within a pH range of 5.0–10.0 with 0.5 pH unit increments. The effect of pH on growth was tested using LB medium (Merck KGaA, Germany) buffered with citric acid (pH 5.0–6.0), phosphate buffer (pH 6.5–8.0), and glycine–NaOH buffer (pH 8.5–10.0). Cultivation analyses for salinity and pH preference were performed at 25 °C. Growth under different temperature, salinity, and pH conditions was assessed by measuring optical density at 600 nm after 48 h of incubation and all cultivation tests were performed in triplicates.

Motility was inspected using the hanging-drop method with a 1000x objective lens with oil immersion and light microscope Motic T025A (Motic, Xiamen, China) after 12 h of incubation in TSB medium supplemented with 5.0% of NaCl (*w/v*). The catalase test was performed using 3% H_2_O_2_ (*w/v*) (CentralChem, Slovakia), and the cytochrome *c* oxidase activity was assessed using oxidase strips (Merck KGaA, Germany). Hydrogen sulfide (H₂S) production was tested using triple sugar iron agar (TSI, Merck KGaA, Germany) supplemented with 5.0% NaCl (*w/v*). Growth without oxygen was tested using an anaerobic jar, ensuring anaerobic conditions with Oxoid AnaeroGen 3.5 L (Thermo Fisher Scientific, Netherlands) on R2A medium supplemented with 5.0% of NaCl (*w/v*), and evaluated after 5 days of cultivation. The nitrate reduction activity was assessed by the Nitrate Reagent Disks Kit (Merck KGaA, Germany) under anaerobic conditions on a nitrate agar, composed of 5.0 g/L peptone, 3.0 g/L yeast extract, and 15.0 g/L agar (all manufactured by Sigma-Aldrich, Germany), 1.0 g/L potassium nitrate and 50.0 g/L NaCl (both CentralChem, Slovakia).

The enzymatic activities, utilization of carbohydrates, alcohols, organic acids, and amino acids were analysed using API^®^ ZYM, API^®^ 50CH kits (bioMérieux, France), and Biolog GEN III MicroPlate™ assays. Cell suspensions were prepared from fresh cultures grown on R2A medium supplemented with 5.0% NaCl (*w/v*), and all inoculation media and suspension solutions were supplemented with 5.0% NaCl (*w/v*) to maintain halophilic conditions.

For fatty acids analysis, strain HP20-50^T^ was cultivated in Marine broth medium (HiMedia, India) or R2A Broth medium (HiMedia, India) supplemented with 5.0% of NaCl (*w/v*) at laboratory temperature (25 °C) for 24 h. The fatty acids analysis was performed at the Identification Service of the DSMZ (Germany) using GC-MS on Agilent 7000D GC-MS (Agilent Technologies, USA).

### Phylogenetic and genomic analyses

Genomic DNA of strain HP20-50^T^ was extracted from overnight culture using a GenElute Bacterial Genomic DNA Kit (Sigma-Aldrich, Burlington, USA), following the manufacturer’s instructions. To ascertain the phylogenetic relationships of strain HP20-50^T^, the 16S rRNA gene was amplified with fD1 (5′-AGAGTTTGATCCTGGCTCAG-3′) and rP2 (5′-ACGGCTACCTTGTTACGACTT-3′) primer set (Weisburg et al. 1991). The cycling conditions and the reaction mixture were used as in Nosalova et al. (2021). The amplicons obtained were then purified using SAP Exokit (Jena Bioscience, Germany) and sequenced using Sanger sequencing method at Eurofins Genomics (Germany). The obtained sequences were processed using the MEGA v12 software (Kumar et al. 2024). The almost complete 16 S rRNA gene sequence was then compared with the sequences available in GenBank database (https://www.ncbi.nlm.nih.gov/genbank/) using blastn tool and deposited under the accession number PP717852.2. A phylogenetic tree was reconstructed using the maximum likelihood method implemented in MEGA v12 (Kumar et al. 2024) with the Kimura 2-parameter model and 1000 bootstrap replicates. The 16 S rRNA gene sequence of *Alteromonas macleodii* was used as an outgroup.

The genome of the strain HP20-50^T^ was sequenced at the Eurofins Genomics Europe Sequencing GmbH (Konstanz, Germany) company using Illumina NovaSeq6000 sequencing and processed as previously described by Brestovičová et al. (2024). In brief, sequence quality was assessed using the FastQC v0.11.9 and sequences were trimmed by Trimmomatic v0.39 (minimum average quality score ≥ 20), both implemented in Unipro UGENE v35.0 software (Okonechnikov et al. 2012; Golosova et al. 2014). Then the genome was de novo assembled by SPAdes v3.12.0 tool (Prjibelski et al. 2020) using high-quality paired-end reads longer than 200 bp with default settings. Genome quality was assessed using CheckM v1.2.3 available on NCBI (Parks et al. 2015). The genome completeness and contamination were estimated to be 98.41% and 1.23%, respectively. The whole-genome sequence has been deposited in the GenBank database under the accession number JAVFHZ000000000.

To determine the phylogenomic position of strain HP20-50^T^, whole-genome sequences of all available type strains of species within the family *Idiomarinaceae* were retrieved from the GenBank database and compared with the genome of strain HP20-50^T^. A maximum-likelihood phylogenomic tree was reconstructed using the EasyCGTree pipeline with default parameters and 1000 bootstrap replicates (Zhang et al. 2023). The resulting Newick tree file was visualised and edited using MEGA v12 (Kumar et al. 2024), and *Alteromonas macleodii* genome was used as an outgroup.

Genome annotation and comparative genome analyses were performed by the Rapid Annotation using Subsystem Technology (RAST) (Aziz et al. 2008; Overbeek et al. 2014; Brettin et al. 2015). Digital DNA-DNA hybridization (dDDH) values were assessed using the Genome-to-Genome Distance Calculator (GGDC) v3.0 (formula 2: identities/HSP length) (Meier-Kolthoff et al. 2022). The average nucleotide identity (ANI) estimation was performed using FastANI (EDGAR platform) (Jain et al. 2018), and the average amino acid identity (AAI) values were calculated using the EDGAR platform (Dieckmann et al. 2021).

The metabolic reconstruction of the genome was assessed using the SEED tool implemented in RAST and compared with the closest representatives of strain HP20-50^T^, with a focus on the pathways involved in stress adaptation to extreme, high-salinity environment.

## Results and discussion

### Cell morphology and phenotypic characterization

**Fig. 1:**
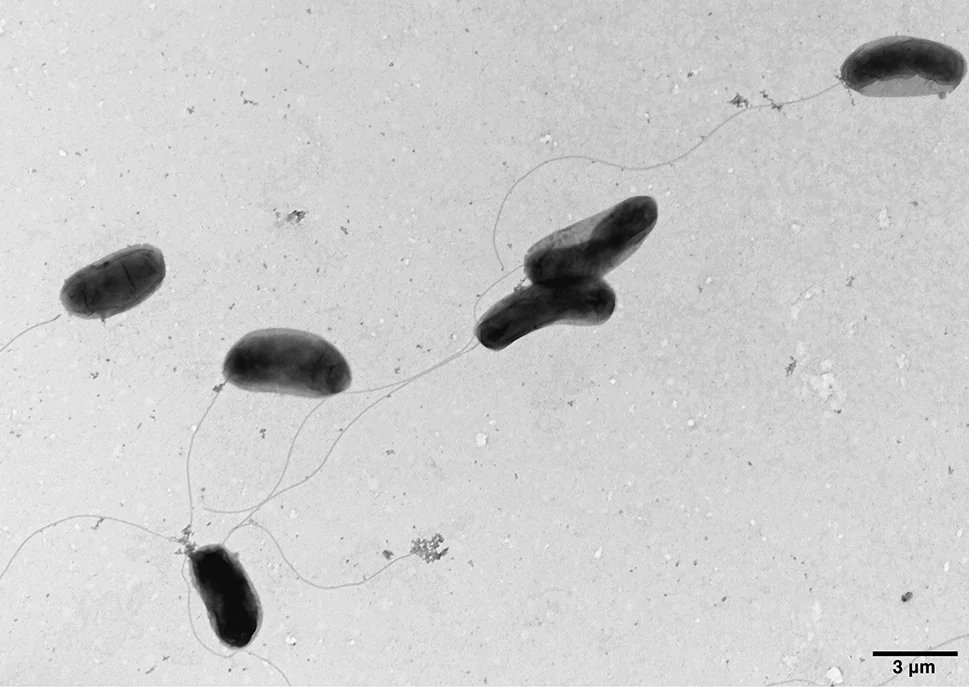
Transmission electron micrograph of strain HP20-50^T^. Cells were negatively stained. Scale bar, 3.0 μm

Cells of strain HP20-50^T^ were Gram-stain-negative, non-endospore-forming short rods (0.9-3.0 × 0.5–0.7 μm), motile by 1–2 polar flagella (Fig. 1). It formed beige and slightly translucent colonies on R2A medium supplemented with 5.0% (w/v) NaCl. The strain was strictly aerobic, catalase- and oxidase-positive, negative for H_2_S production and did not reduce nitrate. Growth was assessed based on visible turbidity after 48 h of incubation. Growth, assessed by optical density measurements at 600 nm (OD_600_) after 48 h of incubation, occurred at 15.0–40.0 °C (optimum 25 °C), at pH 5.0–10.0 (optimum 7.0–7.5), and at salinity ranging from 1.0 to 20.0% (*w/v*) NaCl (optimum 3.0–6.0% (*w/v*). The optimal cultivation conditions for growth were similar to those of the type strains of closely related species (Ivanova et al. 2000; Donachie et al. 2003; Martínez-Cánovas et al. 2004; Li et al. 2022; Hintersatz et al. 2025). The main parameters differentiating strain HP20-50^T^ from the closest related representatives of the genus *Idiomarina* are shown in Table 1.

**Table 1:** Selected phenotypic properties differentiating strain HP20-50^T^ from type strains of closely related species within the genus *Idiomarina*

	1	2	3	4	5	6
Cell morphology	Short rods	Rods	Slightly curved rods	Slightly curved rods	Curved rods	Curved rods
Cell size [µm]	0.9-3.0 × 0.5–0.7	1.0-1.8 × 0.7–0.9	0.7–1.8 × 0.4–0.5	2.0–3.0 × 0.75	0.5–0.8 × 1.5-2.0	2.0–3.0 × 1.5–3.4
Colony pigmentation	Beige, slightly translucent	Non-pigmented	Beige to yellow	Cream	Pale yellow	Beige
Temperature range (optimum) [°C]	15–40 (25)	4–30 (20–22)	4–46 (30)	15–40 (32)	10–45 (37)	4–40 (30)
Salinity range (optimum) [%]	1.0–20.0 (3.0–6.0)	0.6–15.0 (3.0–6.0)	0.5–20.0 (7.0–10.0)	0.5–15.0 (3.0–5.0)	0–20.0 (5.0)	3.0–12.0 (7.0–10.0)
pH range (optimum)	5.0–10.0 (7.0-7.5)	5.5–9.5 (7.0–8.0)	5.5–9.5 (7.5-8.0)	5.0–10.0 (7.0–8.0)	5.0–10.0 (8.0)	6.0–9.0 (7.5-8.0)
Anaerobic growth	-	-	-	-	-	+
Nitrate reduction	-	+	+	-	-	-
H_2_S production	-	-	-	+	+	+
Hydrolysis of:						
Casein	+	-	-	+	+	+
Aesculin	-	-	-	+	+	-
Gelatin	-	-	+	+	+	+
DNA	-	+	+	+	-	+
Utilization of:						
Maltose	+	-	+	-	-	-
Glycerol	-	+	+	-	-	-
Myo-inositol	-	ND	+	ND	-	-
D-glucose	+	-	+	ND	-	-
Acetic acid	-	+	+	-	ND	+
Citric acid	-	-	+	-	ND	-
Lactic acid	-	-	+	-	ND	-
Propionic acid	+	+	+	-	ND	+
L-aspartic acid	-	-	-	ND	ND	+
L-glutamic acid	-	-	+	ND	ND	+
L-alanine	-	+	+	-	-	+
L-serine	-	-	+	-	-	+
β-hydroxybutyric acid	-	-	-	+	ND	+
Major cellular fatty acids	C_11:0_ iso 3-OH	C_15: 0_ iso	C_15: 0_ iso	C_15: 0_ iso	C_15: 0_ iso	C_17:0_ iso
Genomic G + C content [mol%]	46.8	50.0	47.0	46.9	46.8	46.6

Strains: 1, Strain HP20-50^T^ (data from this study);2, *Idiomarina abyssalis* KMM 227^T^ (Ivanova et al. 2000); 3, *Idiomarina loihiensis* L2-TR^T^ (Donachie et al. 2003); 4, *Idiomarina ramblicola* R22^T^ (Martínez-Cánovas et al. 2004); 5, *Idiomarina rhizosphaerae* M1R2S28^T^ (Li et al. 2022); 6, *Idiomarina aminovorans* ATCH4^T^ (Hintersatz et al. 2025)

+ positive, − negative, *ND* no data available

Biochemical characterization revealed positive activity for trypsin and α-chymotrypsin, while weak positive reactions were observed for esterase (C4), esterase lipase (C8), valine arylamidase, cystine arylamidase, and naphthol-AS-BI-phosphohydrolase. The strain was negative for acid phosphatase, α-galactosidase, β-galactosidase, β-glucuronidase, α-glucosidase, and β-glucosidase. Strain HP20-50^T^ utilized L-arginine, propionic acid, and acetic acid, but did not utilize the majority of tested carbohydrates and related compounds, including glucose, fructose, mannose, lactose, sucrose, raffinose, mannitol, glycerol, and several amino acids and organic acids.

### Chemotaxonomic analysis

The cellular fatty acid profile of strain HP20-50^T^ mainly consisted of hydroxy and iso-branched fatty acids (over 50%). The major components were C_11:0_ iso 3-OH (20.1%), C_13:0_ iso 3-OH (15.5%), C_11:0_ iso (10.0%), and C_12:0_ 3-OH (9.0%) (Supplementary Table S1). Moderate amounts of C_10:0_ 3-OH (6.3%) and C_13:0_ iso (5.8%) were also detected. Which is in contrast with closely related *Idiomarina* species, as strain HP20-50^T^ exhibited markedly higher proportions of the hydroxy fatty acids C_11:0_ iso 3-OH and C_13:0_ iso 3-OH (20.1% and 15.5%, respectively), whereas the average proportions of these fatty acids in the reference strains were 5.6% and 3.6%, respectively. In comparison, fatty acids typically dominant in related taxa, such as C_15:0_ iso and C_17:0_ iso, were present in considerably lower amounts in strain HP20-50^T^ (4.3% and 2.6%, respectively) compared with the average values observed in the related strains (26.9% and 13.0%, respectively). Average values were calculated as arithmetic means based on the data for all reference strains included in Supplementary Table S1. This distinctive fatty acid pattern may reflect adaptation to extreme environmental conditions, given that the brine from which the strain was isolated had a relatively higher salt content (~ 311 g/L TDS) compared to seawater (30–45 g/L TDS), a common habitat of *Idiomarina* representatives (Ivanova et al. 2000; Donachie et al. 2003; Martínez-Cánovas et al. 2004; Li et al. 2022). Notably, such a fatty acid composition has not been reported for members of the genus *Idiomarina* or, more broadly, has not been commonly reported in halophilic bacteria. For example, in *Aliidiomarina* sp. 3-OH fatty acids represent 6.1% (Xu et al. 2017) and in *Pseudidiomarina* sp. 7.8% (Jean et al. 2009). Even in *Bacteroidota* sp., known for increased 3-OH fatty acid level, the amount of hydroxylated fatty acids does not exceed 45% and dominant 3-OH fatty acids are C_15_ and C_17:0_ 3-OH acids (Lu et al. 2025). Increased 3-OH fatty acid content in bacterial membranes is more commonly associated with acidophiles and thermophiles, where membrane adaptation plays a crucial role in maintaining stability under stress conditions (Ray et al. 1971; Oshima and Miyagawa 1974; Siristová et al. 2009; Mykytczuk et al. 2010). This fatty acid composition may be linked to membrane-level adaptation to environmental stress at the Solivar site, since stress factors are known to drive remodelling of membrane fatty acids in order to maintain membrane stability and functionality, as was reported in *Pseudomonas syringae* and *Flavobacterium* spp. (Turk et al. 2004; Cray et al. 2015; Králová 2017; García-Descalzo et al. 2023; Maiti et al. 2024). Although cultivation conditions may influence the fatty acid profiles (Mező et al. 2022), the range of differences observed between strain HP20-50^T^ and other *Idiomarina* species cannot be fully explained by cultivation effects alone, given that the same cultivation medium and conditions were used as in the descriptions of closely related type species. In control experiment, using R2A medium supplemented with 5.0% of NaCl (*w/v*), a similar predominance of 3-OH fatty acids was observed (Supplementary Table S2). Therefore, these marked differences more likely reflect the strain’s evolutionary adaptation to its native hypersaline environment.

### Phylogenetic and genomic analyses

**Fig. 2:**
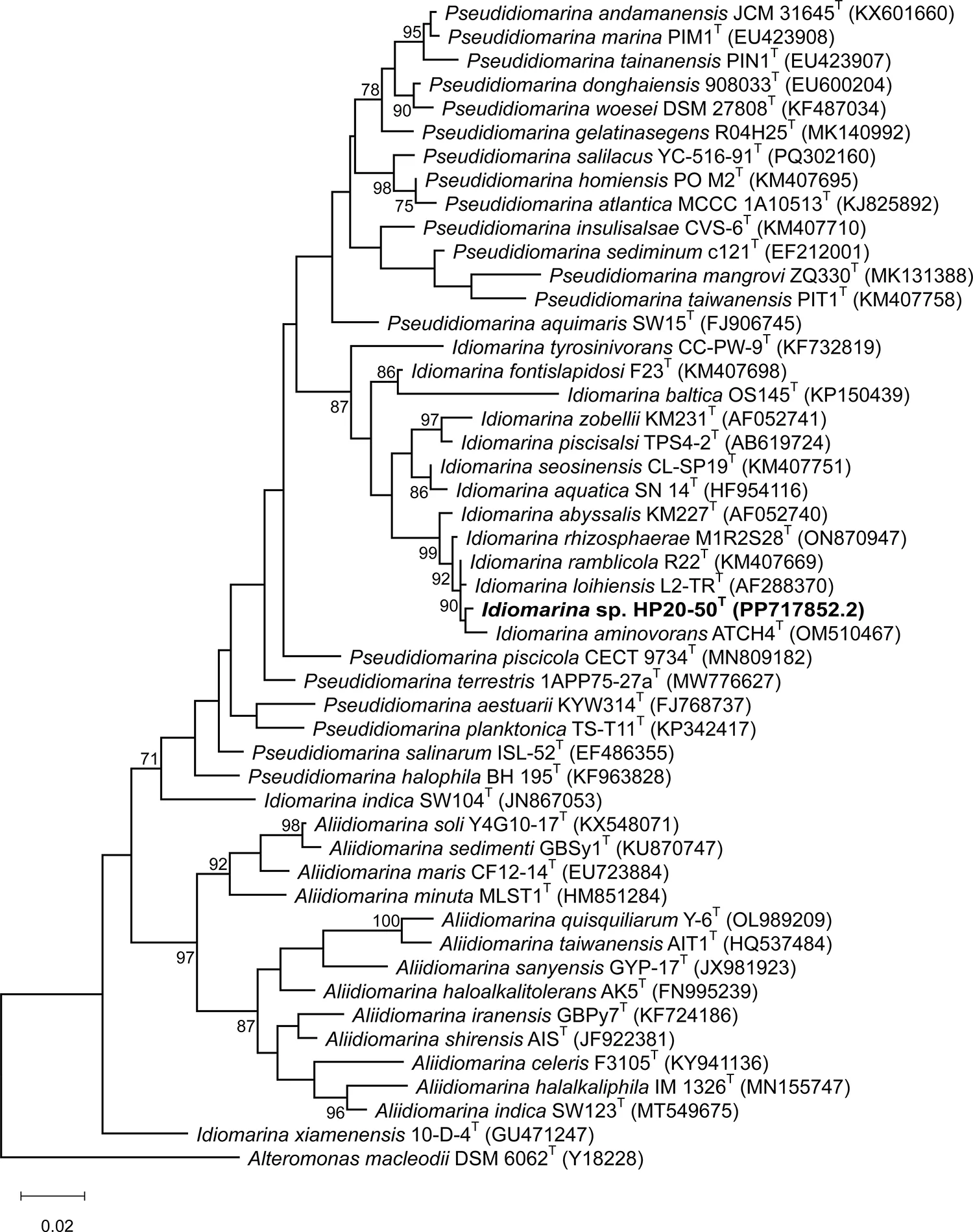
Phylogenetic tree based on 16 S rRNA gene sequences showing the position of strain HP20-50^T^ within the family *Idiomarinaceae.* The phylogenetic tree was constructed using the Maximum likelihood method and the Kimura 2-parameter model. Bootstrap values ≥ 70% calculated based on 1000 bootstrap replications are shown at branch nodes. The 16 S rRNA gene sequence of *Alteromonas macleodii* was used as an outgroup

Comparison of the 16 S rRNA gene sequence of strain HP20-50^T^ with the GenBank database clearly showed that the respective strain belongs to the genus *Idiomarina*, with the highest similarity to *Idiomarina ramblicola* R22^T^ (99.6%), followed by *Idiomarina loihiensis* L2TR^T^ (99.5%), *Idiomarina rhizosphaerae* M1R2S28^T^ (99.2%), *Idiomarina aminovorans* ATCH4^T^ (99.1%), and *Idiomarina abyssalis* KMM 227^T^ (98.8%). This was confirmed by the maximum likelihood phylogenetic tree, based on the 16 S rRNA gene sequences of type strains of the family *Idiomarinaceae*, in which strain HP20-50^T^ clustered within the *I. loihiensis*-related cluster, supported with high bootstrap values (Fig. 2), with *I. aminovorans* as the most related type species. Nevertheless, that the observed similarity values were close to the well-accepted threshold for the species delineation (98.7%; Kim et al. 2014), high resolution analyses (such as genomic analyses) are recommended when providing classification within the family *Idiomarinaceae* (Liu et al. 2018), since 16 S rRNA gene analysis is not reliable marker when used solely.

Therefore, the whole-genome sequencing of strain HP20-50ᵀ was performed, which generated 6,595,060 raw reads. The genome has a total size of 2.80 Mbp with an average sequencing coverage of approximately 660× with 98.41% completeness, N50 value of 374.2 kb and contamination level at 1.23% according to CheckM analysis of the NCBI database. The genomic DNA G + C content is 46.8 mol%, which falls within the range reported for members of the genus *Idiomarina* (Table 2).

**Table 2:** Genome features of strain HP20-50^T^ and *Idiomarina* type strains according to the RAST server and GenBank database

	Strain HP20-50^T^	Idiomarina aminovorans ATCH4^T^	Idiomarina ramblicola R22^T^	Idiomarina rhizosphaerae M1R2S28^T^	Idiomarina loihiensis L2TR^T^	Idiomarina abyssalis MSP-CT^T^	Idiomarina aquatica SN-14^T^	Idiomarina piscisalsi TPS4-2^T^	Idiomarina seosinensis CL-SP19^T^	Idiomarina tyrosinivorans CC-PW-9^T^	Idiomarina zobellii KMM 231^T^	Idiomarina baltica OS145^T^	Idiomarina fontislapidosi CECT5859^T^	Idiomarina xiamenensis 10-D-4^T^
Genome size (Mbp)	2.80	2.75	2.71	2.87	2.84	2.61	2.97	2.58	2.69	2.43	2.58	2.77	2.86	2.90
GC content (%)	46.8	46.6	46.9	46.8	47.0	47.1	51.0	47.0	47.3	49.3	47.1	47.3	47.8	49.5
N50 (kb)	374.2	418.1	361.7	515.0	2839.2	2610.6	1484.6	352.9	594.0	271.2	169.2	89.6	149.4	149.5
L50	3	3	3	3	1	1	1	2	2	4	5	9	5	7
Subsystems	282	277	278	281	286	279	286	275	279	265	275	275	282	273
Coding sequences	2671	2687	2558	2713	2685	2463	2732	2486	2522	2330	2544	2606	2681	2767
RNAs	63	63	49	54	68	68	57	52	56	56	50	49	55	46
Pseudogenes	9	34	6	6	12	2	18	11	10	7	39	38	24	44

The relatively small genome size of strain HP20-50^T^ is consistent with the characteristic genome reduction, observed in genus *Idiomarina*. The genome size in the *Idiomarina* sp. typically does not exceed 3 Mbp, and is significantly smaller than that of other genera within the order *Alteromonadales* (4.0–7.6 Mbp) (Qin et al. 2019), highlighting also the importance of genome analysis when classifying *Idiomarinaceae* representatives. This genome reduction is related to ecological specialization, particularly the preference for protein substrates over carbohydrates, which represents an adaptive strategy for nutrient-poor environments (Qin et al. 2019), such as marine and hypersaline habitats. Only nine pseudogenes were identified, accounting for approximately 0.34% of coding sequences. In prokaryotes, pseudogenes are reported to represent approximately 1–5% of genomes (Liu et al. 2004). This relatively low proportion of pseudogenes observed in the genome of strain HP20-50ᵀ suggests a stable, compact genome organization consistent with long-term ecological specialization (Qin et al. 2019).

Despite the respective strain showed high 16 S rRNA gene similarities (~ 99%) with the type species of the genus *Idiomarina*, comparative genome analyses clearly distinguished strain HP20-50^T^ as a separate species within the genus *Idiomarina*. We observed low relatedness to the closest *Idiomarina* species, with ANI values ranging from 65.0% to 82.8%, AAI values from 67.1% to 91.2%, and digital DNA–DNA hybridization (dDDH) values from 18.2% to 23.2% (Supplementary Table S3). These values are below the generally accepted species threshold of 96% (ANI, AAI) and 70% (dDDH) (Chun et al. 2018), providing strong genomic evidence that strain HP20-50^T^ represents a novel species within the genus *Idiomarina*.

**Fig. 3:**
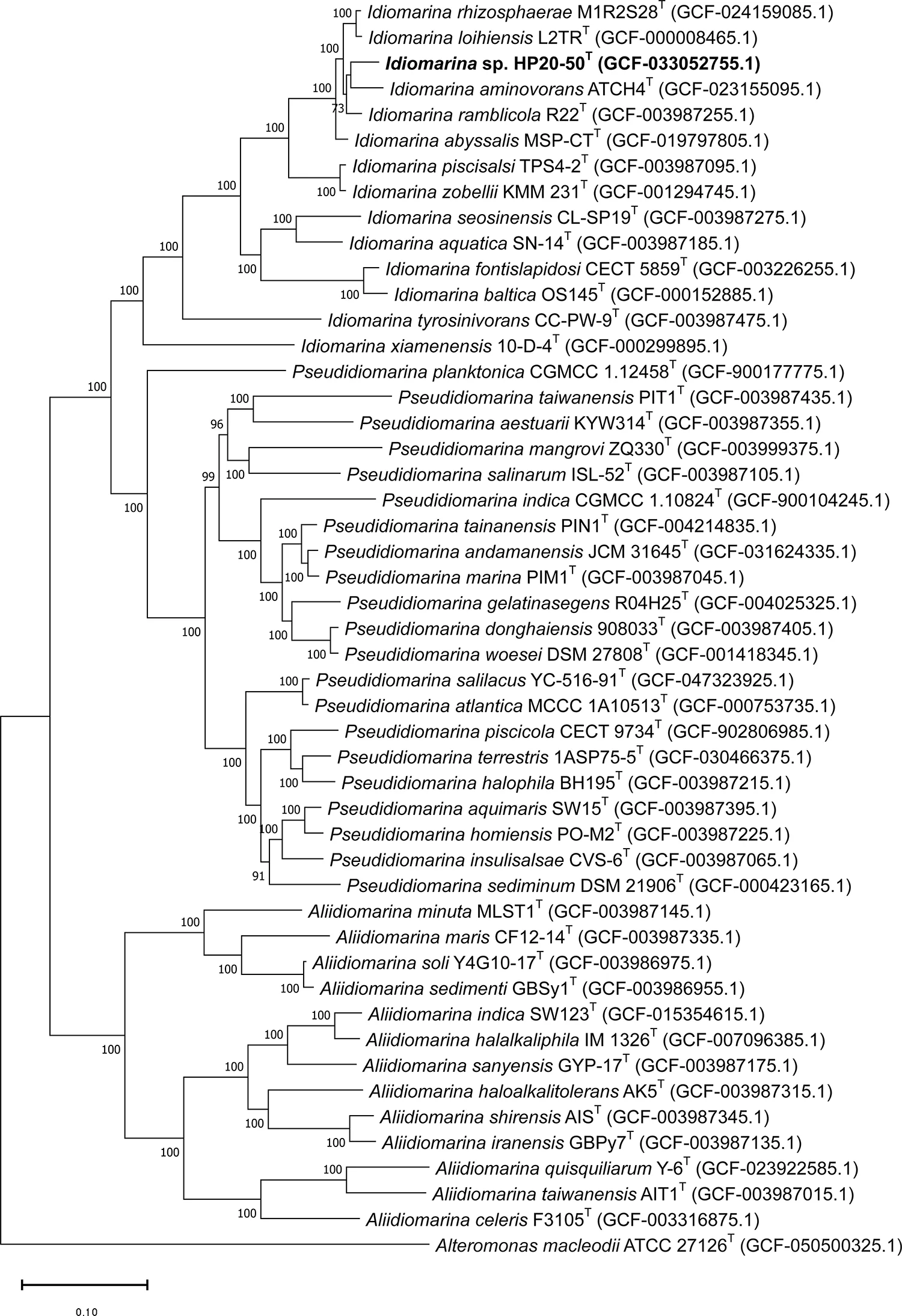
Maximum-likelihood phylogenomic tree showing the position of strain HP20-50^T^ among type strains of the family *Idiomarinaceae* available in the GenBank database, with 1000 bootstrap replications. *Alteromonas macleodii* was used as an outgroup. Only bootstrap values ≥ 70% are shown at branch nodes

Consistent with the comparative genome analysis, the phylogenomic tree placed strain HP20-50^T^ within the genus *Idiomarina*. The strain formed a distinct branch within a well-supported clade comprising *I. aminovorans*, *I. ramblicola*,* I. loihiensis*,* I. rhizosphaerae* and *I. abyssalis* type strains (Fig. 3).

### Genomic adaptation to extreme conditions

Functional genome analysis further revealed several distinctive adaptive traits of strain HP20-50ᵀ. One of the mechanisms of microbial adaptation to high salinity condition, is regulation of intracellular sodium concentration (Gunde-Cimerman et al. 2018). The genome of strain HP20-50^T^ encoded several Na⁺/H⁺ antiporters from the Nha family (NhaC, NhaD, NhaP2) and Na⁺/H⁺ antiporters subunit A, B, C, D, E, F, G, suggesting an active sodium export system that maintains ion balance in the cytoplasm under conditions of high NaCl content. In addition, the presence of a potassium efflux system (KefA) further supports regulated control of intracellular potassium concentrations, contributing to overall ion homeostasis under osmotic stress (Rasmussen 2023; Kaur and Kaur 2024). This system appears to be widely conserved within the genus, as it was identified in all *Idiomarina* strains examined by Kaur and Kaur (2024), while it was not detected in the recently described *Idiomarina aminovorans* (Hintersatz et al. 2025). Compatible osmoadaptation based on soluble substances is supported by the presence of the *betB* gene encoding betaine aldehyde dehydrogenase, indicating the ability to biosynthesize glycine betaine. Glycine betaine is a well-known osmoprotectant in mesohalophilic bacteria, and contributes to the stabilization of proteins and cell structures without affecting enzymatic processes (Imhoff 2020). Such compatible solutes may therefore support metabolic activity under osmotic stress, which is consistent with reports of stable and active enzymes produced by the genus *Idiomarina* (Zhou et al. 2018), despite they were not observed in the *I. loihiensis* and *I. rhizosphaerae* (Supplementary Figure S1).

The genome also contained an extensive periplasmic and envelope stress response network. Identified genes include genes for sigma factor RpoE and its negative regulators RseA and RseB, together with the outer membrane stress sensor proteases DegS and DegQ. Additionally, genes for intramembrane protease RasP/YluC and the outer membrane protein H precursor were detected. These components form part of the σE-dependent envelope stress response system, which is crucial for maintaining outer membrane integrity under osmotic and environmental stress conditions (Raivio 2005; Ades 2008). Genes associated with resistance to heavy metals were also detected, in particular for copper resistance (*copC*, *copD*). All these genomic features, previously described in details by Brestovičová et al. (2024), suggest specific ecological adaptations, and further support the uniqueness of strain HP20-50^T^. Notably, the distinctive fatty acid profile observed in strain HP20-50^T^ further complements these genomic traits and may represent an additional level of adaptation to hypersaline conditions. This combination of membrane and genomic traits expands the current understanding of the molecular mechanisms underlying adaptation to high salinity conditions and requires further detailed investigation.

Based on the results of polyphasic analysis presented here, we conclude that strain HP20-50^T^ represents a novel species of the *Idiomarina* genus, for which the name *Idiomarina solivarensis* sp. nov. is proposed.

### Description of *Idiomarina solivarensis* sp. nov.

*Idiomarina solivarensis* (so.li.va.ren’sis N.L. fem. adj. solivarensis, referring to salt mine in Solivar, Slovakia, where the type strain was isolated).

Cells are Gram-negative, strictly aerobic and motile short rods. When cultivated on R2A medium supplemented with 5.0% of NaCl (*w/v*), colonies are beige and slightly translucent. Obligate halophile, catalase- and oxidase-positive, negative for H_2_S production and does not reduce nitrate. Growth occurs at 15–40 °C (optimum 25 °C), at pH 5–10 (optimum pH 7.0–7.5), and in the presence of 1.0–20.0% (*w/v*) NaCl, with an optimal growth at 3.0–6.0% of NaCl (*w/v*). The dominant fatty acids are C_11:0_ iso 3-OH, C_13:0_ iso 3-OH, C_11:0_ iso, and C_12:0_ 3-OH. Positive activity for trypsin and α-chymotrypsin on API^®^ZYM. Negative reactions are observed for acid phosphatase, α-galactosidase, β-galactosidase, β-glucuronidase, α-glucosidase, and β-glucosidase. Positive for utilization of L-arginine, propionic acid, and acetic acid, but negative for the following carbohydrates and organic compounds (assessed by Biolog GEN III and API^®^50CH): dextrin, D-maltose, D-trehalose, D-cellobiose, gentiobiose, sucrose, D-turanose, stachyose, D-raffinose, α-D-lactose, D-melibiose, β-methyl-D-glucoside, D-salicin, *N*-acetyl-D-glucosamine, *N*-acetyl-D-mannosamine, *N*-acetyl-D-galactosamine, *N*-acetyl-neuraminic acid, α-D-glucose, D-fructose, D-mannose, D-galactose, 3-methyl glucose, D-fucose, L-fucose, L-rhamnose, inosine, D-sorbitol, D-mannitol, D-arabitol, *m*-inositol, glycerol, α-D-lactose, lactulose, D-glucose-6-phosphate, D-fructose-6-phosphate, D-aspartic acid, D-serine, gelatin, glycyl-L-proline, L-alanine, L-aspartic acid, L-glutamic acid, L-histidine, L-pyroglutamic acid, L-serine, pectin, D-galacturonic acid, L-galactonic acid lactone, D-gluconic acid, D-glucuronic acid, glucuronamide, mucic acid, quinic acid, D-saccharic acid, *p*-hydroxy-phenylacetic acid, methyl pyruvate, D-lactic acid methyl ester, L-lactic acid, citric acid, α-keto-glutaric acid, D-malic acid, L-mallic acid, bromosuccinic acid, tween 40, γ-amino-butyric acid (GABA), α-hydroxy-butyric acid, β-hydroxy-D, L-butyric acid, α-keto-butyric acid, acetoacetic acid.

The type strain is HP20-50^T^ (= DSM 120110^T^ =CCM 9475^T^) isolated from the brine of the former salt mine in Solivar (Slovakia). The genomic G + C content of the type strain is 46.8%. The GenBank accession numbers for the 16 S rRNA gene sequence and genome are PP717852.2 and JAVFHZ000000000, respectively.

## Supplementary Information

Below is the link to the electronic supplementary material.


Supplementary Material 1


## Data Availability

The 16 S rRNA gene sequence and the whole-genome sequence analysed during the study are available in the GenBank database under the accession numbers: PP717852.2, and JAVFHZ000000000, respectively. All data supporting the findings of this study are available within the paper and its Supplementary Information.
