# Supplementary material for: Idiomarina solivarensis sp. nov., halophilic bacterium isolated from brine of the former subsurface salt mine in Solivar (Slovakia) showing a unique fatty acids profile
Source: Arch Microbiol. 2026 Jun 16;208(9):439. doi: 10.1007/s00203-026-05004-3 (PMC13272253; doi:10.1007/s00203-026-05004-3)

**Supplementary Table S1** The main differences in cellular fatty-acid composition (%) of strain HP20-50^T^ and closely related *Idiomarina* species

Strains: 1, Strain HP20-50^T^ (data from this study); 2, *Idiomarina abyssalis* KMM 227^T^ (Ivanova et al. 2000); 3, *Idiomarina loihiensis* L2-TR^T^ (Donachie et al. 2003); 4, *Idiomarina ramblicola* R22^T^ (Martínez-Cánovas et al. 2004); 5, *Idiomarina rhizosphaerae* M1R2S28^T^ (Li et al. 2022); 6, *Idiomarina aminovorans* ATCH4^T^ (Hintersatz et al. 2025)

|  | 1^*^ | 2 | 3 | 4 | 5 | 6 |
| --- | --- | --- | --- | --- | --- | --- |
| C_10:0_3-OH | 6.3 | ---- | ---- | 1.1 | 1.2 | ---- |
| C_11:0_iso | 10.0 | ---- | 2.0 | 3.4 | 2.9 | 4.0 |
| C_11:0_ iso 3-OH | 20.1 | ---- | 4.1 | 5.6 | 7.4 | 5.5 |
| C_12:0_ 3-OH | 9.0 | ---- | ---- | ---- | 1.2 | 1.6 |
| C_13: 0_ iso | 5.8 | 1.0 | 1.8 | 1.5 | 2.4 | 2.2 |
| C_13: 0_iso 3-OH | 15.5 | ---- | 3.3 | 2.3 | 3.4 | 5.3 |
| C_15: 0_anteiso | TR | ---- | ---- | 1.2 | ---- | ---- |
| C_15: 0_iso | 4.3 | 33.7 | 32.6 | 24.7 | 19.8 | 23.8 |
| C_16: 1_*ω7c* | ---- | 7.0 | 6.0 | 5.2 | ---- | ---- |
| C_17: 1_*ω*7c iso | ---- | ---- | 11.9 | 11.0 | ---- | ---- |
| C_17: 0_iso | 2.6 | 11.9 | 11.0 | 12.9 | 11.7 | 17.7 |
| C_17: 0_cyclo | TR | ---- | 1.7 | 2.5 | ---- | 11.5 |
| C_18: 1_*ω*7c | ---- | 6.7 | 5.5 | 5.9 | ---- | ---- |
| C_19:0_cyclo *ω*7c | ---- | ---- | ---- | ---- | ---- | 7.1 |

*Data from this study

TR, trace amounts <1%

|  | Marine Broth + 5.0% NaCl | R2A +5.0% NaCl |
| --- | --- | --- |
| C_10:0_3-OH | 6.3 | 9.4 |
| C_11:0_iso | 10.0 | 10.9 |
| C_11:0_ iso 3-OH | 20.1 | 13.4 |
| C_12:0_ 3-OH | 9.0 | 9.0 |
| C_13: 0_ iso | 5.8 | 4.2 |
| C_13: 0_iso 3-OH | 15.5 | 9.0 |
| C_15: 0_anteiso | 0.2 | 0.1 |
| C_15: 0_iso | 4.3 | 4.5 |
| C_16: 0_ | 3.7 | 6.2 |
| C_17: 0_iso | 2.6 | 2.1 |
| C_17: 0_cyclo | 0.2 | 0.3 |
| C_18: 0_ | 1.0 | 1.4 |

**Supplementary Table S2** Comparison of cellular fatty acid composition (%) of strain HP20-50^T^ cultivated in two different media

**Supplementary Table S3** *In silico* DNA-DNA hybridisation (dDDH), average nucleotide identity (ANI), and average amino acid identity (AAI) values of strain HP20-50^T^ compared with the representatives of the genus *Idiomarina*

| Reference genome | DDH (%) | ANI (%) | AAI (%) |
| --- | --- | --- | --- |
| *Idiomarina abyssalis* MSP-CT^T^  (GCA_019797805.1) | 22.7 | 82.6 | 90.9 |
| *Idiomarina aquatica* SN-14^T^  (GCA_003987185.1) | 19.3 | 79.5 | 75.0 |
| *Idiomarina baltica* OS145^T^  (GCA_000152885.1) | 20.3 | 65.0 | 72.3 |
| *Idiomarina fontislapidosi* CECT5859^T^  (GCA_003226255.1) | 18.2 | 65.0 | 72.3 |
| *Idiomarina loihiensis* L2TR^T^  (GCA_000008465.1) | 23.1 | 82.8 | 90.6 |
| *Idiomarina piscisalsi* TS4-2^T^  (GCA_003987095.1) | 18.3 | 79.6 | 82.7 |
| *Idiomarina ramblicola* R22^T^  (GCA_003987255.1) | 23.2 | 82.7 | 91.2 |
| *Idiomarina rhizosphaerae* M1R2S28^T^  (GCA_024159085.1) | 22.8 | 82.4 | 90.7 |
| *Idiomarina seosinensis* CL-SP19^T^  (GCA_003987275.1) | 19.2 | 80.3 | 74.9 |
| *Idiomarina tyrosinivorans* CC-PW-9^T^  (GCA_003987475.1) | 18.7 | 65.0 | 69.1 |
| *Idiomarina xiamenensis* 10-D-4^T^  (GCA_000299895.1) | 20.7 | 65.0 | 67.1 |
| *Idiomarina zobellii* KMM 231^T^  (GCA_900100855.1) | 18.7 | 80.2 | 82.7 |
| *Idiomarina aminovorans* ATCH4^T^  (GCA_023155095.1) | 22.9 | 81.9 | 90.5 |

**Supplementary Figure S1** Comparison of metabolic reconstruction analysis results of the strain HP20-50^T^ with the closest *Idiomarina* representatives, with a focus on adaptation to extreme high-salinity conditions


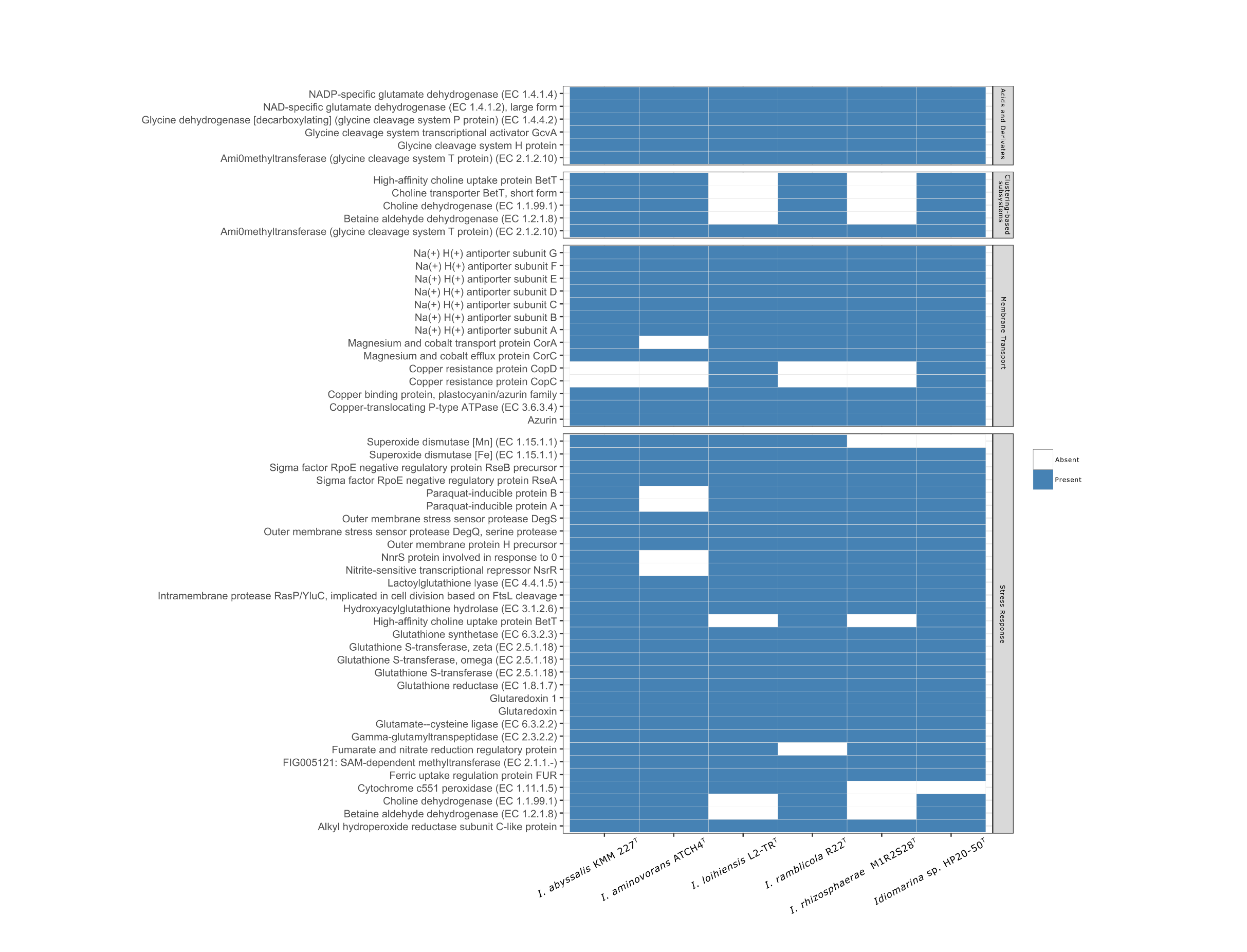

Supplement: Supplementary file 1 — Supplementary Material 1 [file 203_2026_5004_MOESM1_ESM.docx]
